# A holistic approach to determine tree structural complexity based on laser scanning data and fractal analysis

**DOI:** 10.1002/ece3.3661

**Published:** 2017-11-23

**Authors:** Dominik Seidel

**Affiliations:** ^1^ Silviculture and Forest Ecology of the Temperate Zones Faculty of Forest Sciences University of Göttingen Göttingen Germany

**Keywords:** competition, complexity, fractal analysis, LiDAR, management, shape, structure, three‐dimensional, tree architecture

## Abstract

The three‐dimensional forest structure affects many ecosystem functions and services provided by forests. As forests are made of trees it seems reasonable to approach their structure by investigating individual tree structure. Based on three‐dimensional point clouds from laser scanning, a newly developed holistic approach is presented that enables to calculate the box dimension as a measure of structural complexity of individual trees using fractal analysis. It was found that the box dimension of trees was significantly different among the tested species, among trees belonging to the same species but exposed to different growing conditions (at gap vs. forest interior) or to different kinds of competition (intraspecific vs. interspecific). Furthermore, it was shown that the box dimension is positively related to the trees’ growth rate. The box dimension was identified as an easy to calculate measure that integrates the effect of several external drivers of tree structure, such as competition strength and type, while simultaneously providing information on structure‐related properties, like tree growth.

## 1 INTRODUCTION

Many ecosystem functions and services provided by forests, such as biodiversity (e.g., Lindenmayer, Margules, & Botkin, 2000), productivity (e.g., Ishii, Tanabe, & Hiura, 2004), habitat suitability (Eichhorn et al., 2017; MacArthur & MacArthur, 1961; Tilman & Kareiva, 1997), or recreational benefit (e.g., Ribe, 2009) as well as ecosystem resilience and adaptability (McElhinny, Gibbons, Brack, & Bauhus, 2005; Neill & Puettmann, 2013; Schulze, Beck, & Müller‐Hohenstein, 2002), are affected by forest structure. Despite this great importance, very little is known about forest structure in all three dimensions and how it is naturally formed and artificially altered. The enormous complexity, size, and diversity of forests structures only allowed for a rudimentary assessment in the past (cf. review: Seidel, Fleck, Leuschner, & Hammett, 2011). Tomlinson (1983) argued that “it seems inherently reasonable to approach an understanding of how forests are made by finding out how individual units of the forest—the trees themselves—develop”. A closer look at tree individuals may hence help to understand the forest structure as a higher unit of organization.

The architecture of a tree is the result of a stochastic growth process. It is, however, not entirely random, as genetics (cf. Hallé & Oldeman, 1970) as well as environmental factors, such as aboveground competition (e.g., Bayer, Seifert, & Pretzsch, 2013; Seidel, Leuschner, Müller, & Krause, 2011), wind (e.g., Brüchert & Gardiner, 2006), water availability (e.g., Archibald & Bond, 2003), and others determine the architecture of a tree to some degree. In the past, architectural models, such as those presented by Hallé, Oldeman, or Tomlinson (Hallé & Oldeman, 1970; Hallé, Oldeman, & Tomlinson, 1978; Tomlinson, 1983), were used to describe general principles of the construction of trees. These principles were formulated as “precise genetic ground plan[s]” (Tomlinson, 1983) and describe the architecture of a tree mostly based on growth dynamics and branching pattern, such as sympodial versus monopodial growth. Such principles are independent from size and discovering them facilitated research focusing on the relationship between tree function and form (Tomlinson, 1983).

In addition to such “architectural” approaches, one may assess tree structure using attributes like crown volume (Moorthy et al., 2011), crown surface area (Metz et al., 2013), tree height (Seidel, Leuschner et al., 2011), taper, lean and sweep of the stem (e.g., Thies, Pfeifer, Winterhalder, & Gorte, 2004), or crown radius (Seidel, Schall, Gille, & Ammer, 2015). Such morphological measures describe the growth habit, tree form or the shape of specific tree elements, or complete individuals. Related research mostly focused on investigating alternations in tree shapes (phenotypes) in response to external effects, such as different types of competitive interference as induced by mixed or pure neighborhoods (Bayer et al., 2013; Metz et al., 2013; Seidel, Leuschner et al., 2011). Thereby, the focus lay on which elements of a tree changed in shape or geometry (e.g., branching angle and branch length), rather than how the element's geometries were created by “genetic growth plans” in the first place. Such work enabled an understanding of the effects of environmental conditions on tree's functions and services, such as carbon sequestration or provision of wood and habitat. Species identity and environmental effects, may it be competition, wind, terrain conditions, management intensity, or others, potentially alter the architectural model of trees. Understanding both, the architectural plan and the effects of environmental conditions seem to be the ultimate goal.

The fractal‐like nature of the crown (and root) system of trees, which is assumed responsible for general allometric relationships and scaling laws (e.g., Duursma et al., 2010; Mandelbrot, 1977; West, Brown, & Enquist, 1999), is one specifically important aspect of tree structure. The father of fractal geometry, Benoît Mandelbrot, emphasized that the nested irregularity of natural objects like trees is a source for simplicity when analyzing complex structures (Mandelbrot, 1977; cf. Sugihara & May, 1990). Addressing structural complexity based on the fractal analysis holds potential to be a tool for characterizing a tree's structure both in terms of “space‐filling” (how much space is occupied by the organs of a tree) and spatial pattern (distribution of organs in space) with a single meaningful measure (e.g., Jonckheere, Nackaerts, Muys, van Aardt, & Coppin, 2006; Kaye, 1994; Zeide & Pfeifer, 1991).

Here, a new holistic approach based on terrestrial laser scanning and fractal analysis is presented that can be used to describe the structural complexity of individual trees. To evaluate the potential of this new application for future research on tree and forest structure, 149 trees available through four laser scanning campaigns previously conducted in the USA (1) and Germany (3) were used.

Based on four hypotheses, it was evaluated whether the so called “box dimension” may be a meaningful measure to distinguish tree shapes that are due to (1) different species identities, (2) different growing conditions (at gap vs. in interior), or (3) different neighborhood diversity (monoculture vs. three neighbor species). Additionally, it was tested whether the box dimension is related to the trees’ growth performance (structure‐function link).

## 2 MATERIALS AND METHODS

### 2.1 Calculation of box dimension

**Figure 1:**
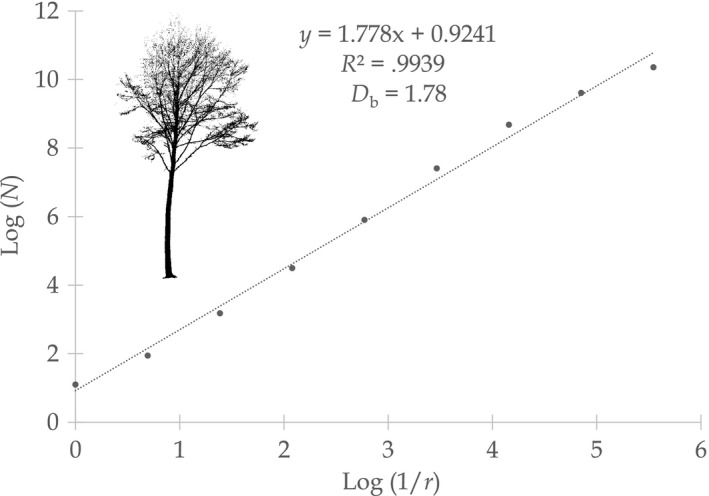
Exemplary log–log plot of the number of boxes [*N*] over the inverse of the box size [*r*] for the point cloud of a beech tree (*Fagus sylvatica* L.; upper left). The tree is growing in the Hainich National Park and is 29.95 m in height. It was scanned with ten terrestrial laser scans and consists of about 205,000 points. The slope of the fitted straight line (1.78) equals the box dimension (*D*_b_) of the tree. Box sizes (edge‐length) ranged from 30 m (left on *x*‐axis) to 10 cm (right on *x*‐axis)

In fractal analysis, the box dimension (*D*_b_), also known as Minkowski–Bouligand dimension, is frequently used to estimate the fractal dimension of objects, and it is considered a holistic measure of structural complexity (Mandelbrot, 1977). Based on a newly developed routine written in Mathematica (Wolfram Research, Champaign, USA), *D*_b_ was derived from laser‐based three‐dimensional point clouds of trees. *D*_b_ was calculated as the slope of the fitted straight line (least square fit) through a plot of log(*N*) over log(1/*r*), with log() being the natural logarithm, and *N* being the number of boxes of size *r* needed to enclose all points in a tree's point cloud (Mandelbrot, 1977; Sarkar & Chaudhuri, 1994). Earlier studies argued that placing tree crowns in boxes “[…] is costly and has technical difficulties in data acquisition” (Zhu, Wang, Chen, Huang, & Yang, 2014). This, however, is not true anymore if virtually conducted using the 3D tree models from laser scanning data. The point cloud of a tree, which is not more than a list of three‐dimensional Cartesian coordinates, is simply converted into what is often named “voxel models” (voxel = (“volumetric pixels” ≈ boxes) of different resolutions, and the number of voxels needed to “cover” the tree's point cloud is counted. Voxel models were used in the past in various studies dealing with laser scanning data (e.g., Cifuentes, Van der Zande, Farifteh, Salas, & Coppin, 2014; Juchheim, Ammer, Schall, & Seidel, 2017; Juchheim, Annighöfer et al., 2017). An example for the calculation of the box dimension of an exemplary tree is presented in Figure 1.

### 2.2 Study objects

#### 2.2.1 Box dimension and species identity

It was tested whether trees of the three species European ash (*Fraxinus excelsior* L.; *n* = 5), European beech (*Fagus sylvatica* L., *n* = 59), and Norway spruce (*Picea abies* L.; *n* = 10) differ in their box dimension due to morphological differences. Study trees of the three species were randomly selected from the dataset presented in Metz et al. (2013; details on the study sites can be found therein) and selected to be taller than 25 m for better comparability. A comparison of the performance of the *D*_b_‐based approach with a conventional approach is not possible in this first part of the analysis as there are no other means for tree species differentiation that is solely based on structural information. Tree species identification from the ground is routinely conducted based on visual assessment by experts.

#### 2.2.2 Box dimension and growing conditions

A dataset of 38 Douglas‐fir trees (*Pseudotsuga menziesii* (Mirb.) Franco), 18 of which growing at the edge of 0.2 ha canopy gaps and 20 growing in the interior of the stand, was chosen to evaluate whether effects of one‐sided competition on tree structure are reflected in the box dimension of the trees. Details on the study sites near Eugene, Oregon (USA), and the tree individuals can be found in Seidel, Ruzicka, and Puettmann (2016). Results from the same study were used to compare the performance of *D*_b_ with the findings of existing laser‐based approaches to measure tree structural changes.

#### 2.2.3 Box dimension and neighborhood diversity

To investigate the effect of neighborhood diversity on target tree structure, a sample of 14 European beech (*Fagus sylvatica* L.) trees growing in unmanaged forests of the UNESCO World heritage site Hainich National Park was used. All trees were taller than 25 m in height. One group of trees (*n* = 7) grew in pure neighborhoods with monospecific competition by beech. The individuals of a second group (*n* = 7) grew in neighborhoods that consisted of three different species (beech + two other). The admixed species were comprised by sycamore maple (*Acer pseudplatanus* L.), lime tree (*Tilia cordata* L.), European ash (*Fraxinus excelsior* L.), and hornbeam (*Carpinus betulus* L.). It was tested whether the box dimension of beech trees growing in pure stands was significantly different from trees growing in mixed neighborhoods. Here, data on the same 14 trees from Juchheim, Annighöfer, et al. (2017) were used to evaluate the performance of *D*_b_ when opposed to existing structural measures that change in dependence on neighborhood diversity.

#### 2.2.4 Box dimension and growth performance

At last, it was tested whether the box dimension may be a predictor of the trees’ growth performance. Therefore, a sample of 23 beech trees that were scanned for a previous study (Metz et al., 2013) was used. Data on the one‐year relative diameter increment (2012) were available from measurements taken from self‐acting DC2 circumference dendrometers (www.ecomatik.de; see Metz et al., 2013 for details). In order to enable a comparison between the box dimension approach and existing approaches, the strength of the relationship with tree growth will be opposed to the findings of Metz et al. (2013) who investigated the relationship between growth performance and competition indices.

### 2.3 Statistics

The free statistical software R (Vers. 3.4, R Development Core Team) was used for all statistical analysis. For the analysis of the effects of species identity on *D*_b_, a one‐way ANOVA with Tukey's post hoc test (two‐sided Welch *t* test) was conducted. To investigate the effects of neighborhood diversity and the growing conditions (gap vs. no gap) on *D*_b_, two‐sided Welch *t* tests were used, and the relationship between tree growth and *D*_b_ was assessed based on Pearson‐correlation analysis.

## 3 RESULTS

### 3.1 Box dimension and species identity

**Figure 2:**
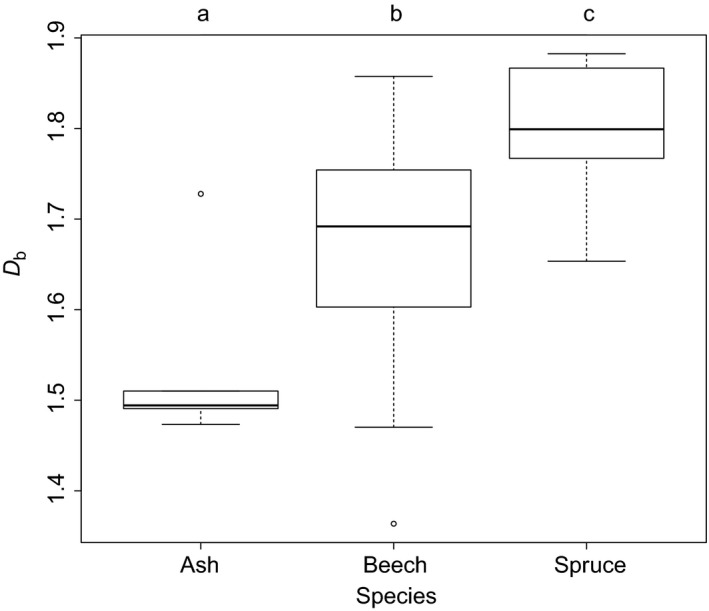
Box and Whisker plots of the box dimension (*D*_b_) for the individuals of the three investigated tree species. Different lowercase letters indicate significant differences between the means at *p* < .05

A differentiation of the three tested species was clearly possible based on the box dimension. Lowest *D*_b_ was measured for ash trees, largest for spruce, and intermediate for beech (Figure 2).

### 3.2 Box dimension and growing conditions

**Figure 3:**
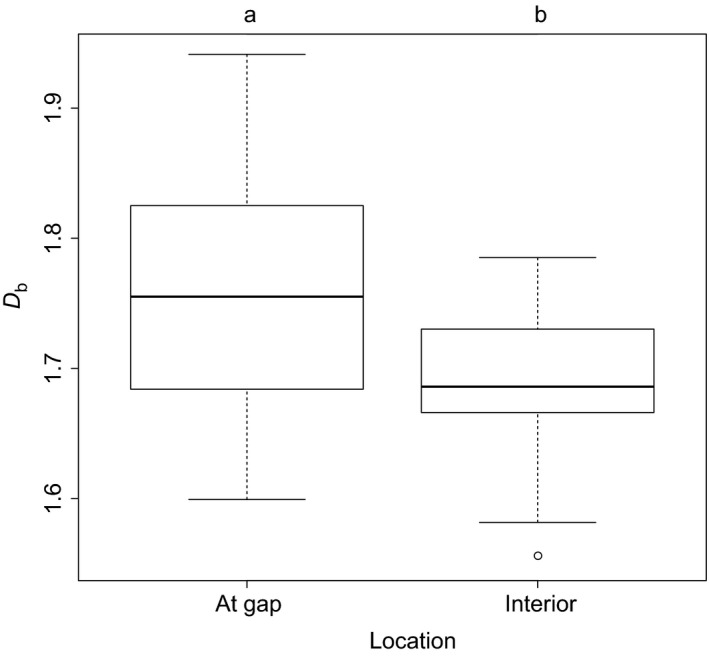
Box and Whisker plots of the box dimension (*D*_b_) for the two groups of Douglas‐Fir trees. Different lowercase letters indicate significant differences between the means at *p* < .05

The Welch *t* test revealed that the box dimension was significantly different between the two groups. Trees growing on the edge of a gap developed more complex crowns than trees growing in the undisturbed interior of the stand (Figure 3). Existing laser‐based approaches applied to the same trees (data from Seidel et al., 2016) also revealed significant differences among the trees for the individual structural measures crown base height (*p* < .001), height of the maximum crown projection area (*p* < .001), live crown ratio (*p* < .001), crown length (*p* < .01), crown surface area (*p* = .01), and crown volume (*p* < .05).

### 3.3 Box dimension and neighborhood diversity

**Figure 4:**
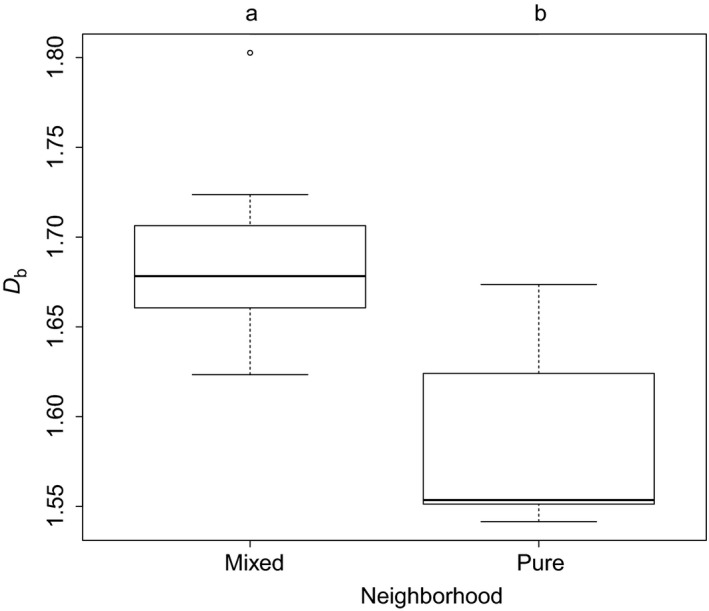
Box and Whisker plots of the box dimension (*D*_b_) for individuals either growing in pure or mixed neighborhoods. Different lowercase letters indicate a significant difference between the means at *p* < .01

It was found that the box dimension was significantly lower for trees growing in pure neighborhoods (intraspecific competition only) than for trees growing in mixed neighborhoods (interspecific competition; see Figure 4). For the 14 trees investigated here, data from Juchheim, Ammer et al. (2017) and Juchheim, Annighöfer et al. (2017) revealed significant differences for the existing laser‐based measures total tree height (*p* < .05), mean length of branches (*p* < .001), and mean branch angle (*p* < .05).

### 3.4 Box‐dimension and growth

**Figure 5:**
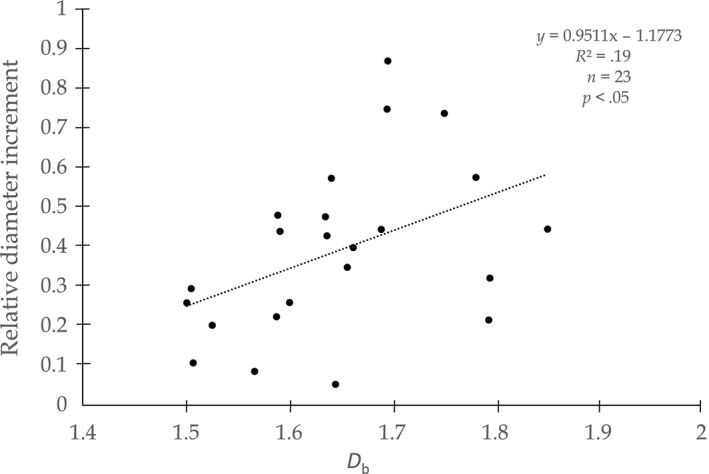
Scatter plot of the relative diameter increment of 23 beech trees as a function of the trees’ box dimension (*D*_b_)

The relative diameter increment for the one‐year measurement period (vegetation period of 2012) significantly increased with the box dimension of the 23 beech trees (Figure 5). In a previous study by Metz et al. (2013) using the same trees, no correlation was found between the conventional competition index KKL (Pretzsch, 1995) and tree growth, but a significant one between the laser‐based competition measures “cumulative crown surface area” (CCSA) and tree growth.

## 4 DISCUSSION

Mandelbrot (1977) presented theoretical considerations suggesting that the fractal dimension of trees, which can be estimated by the box dimension, is smaller than two. He supported his findings with statements from Leonardo da Vinci, who communicated similar thoughts. A high *D*_b_ (maximum of *D*_b_ is three, cf. Mandelbrot, 1977) means the tree has a “space‐filling character,” while a branch‐free “pole” would have a value close to one. Values close to the maximum of three make sense for organisms that intend to maximize the exchange surface with an omnipresent media. For example, the bronchial tree of the lung is “designed” to maximize the exchange between oxygen in the air and the blood (Mandelbrot, 1977). For botanical trees however, it is not the surrounding air, or the carbon dioxide therein, it is the light that is the limited aboveground resource. In order to capture a maximum number of photons, a space‐filling character is of no use due to self‐shading. Therefore, trees seem not to strive for a maximized box dimension close to three but a significantly smaller one (Mandelbrot, 1977). In this study, no tree had a *D*_b_ greater than 1.9, but one has to consider that none of the trees grew in the open.

It appears that since Mandelbrot there has been no significant methodical advancement that would allow determining what the fractal dimension or the box dimension (as an estimate of the fractal dimension) of trees actually is. Previous pioneer work was based on proxies like the ratio between the convex hull volume of the crown and the crown surface area as a measure of fractal dimension of the crown surface (Zeide & Gresham, 1991; Zeide & Pfeifer, 1991). The same studies also showed that the fractal dimension of the crown surface was related to site quality and thinning intensity (Zeide & Gresham, 1991; Zeide & Pfeifer, 1991).

Here, it was hypothesized that the box dimension differs significantly among tree species, growing conditions (gap vs. interior), and local neighborhood composition. The data showed that general differences in *D*_b_ exist among the tested tree species beech, ash, and spruce. Interestingly, the *D*_b_'s of the two‐broadleaved tree species was no more similar than that of beech and the conifer. However, this may be attributed to the rather small sample size. In addition, the investigated tree species are known to be of rather great difference according to their general architecture (e.g., Tomlinson, 1983) and crown shape (e.g., Seidel, Leuschner et al., 2011).

Furthermore, evidence was found that *D*_b_ of trees is indeed related to the existing light regime a tree is exposed to. Douglas‐fir trees growing near gaps had higher *D*_b_ than control trees growing in the interior, as the first had access to more light including incident angles close to the horizon, while the latter received less light and the angle of incidence was limited to zenithal directions. The degree to which a tree is exposed to competition should hence be negatively related to the box dimension as there is no “motivation” (in form of available light) to grow in a “space‐filling” pattern (high *D*_b_). Here, support for this hypothesis was found for beech trees, revealing a higher *D*_b_ when growing in mixture with other tree species than in pure neighborhoods. This is in line with earlier studies, showing that beech develops wider crowns (e.g., Bayer et al., 2013) and experiences less competition pressure if exposed to interspecific competition when compared to intraspecific competition (e.g., Metz et al., 2013). For the sample of beech trees investigated here, Metz et al. (2013) showed that individuals in the mixed neighborhoods were less affected by competition than individuals in pure neighborhoods.

Under a given light regime, trees may develop a crown shape that may be the best adaptation possible under the given environmental condition, for example, available growing space, and that in turn results in a certain box dimension. Consequently, for beech trees, it was shown that the box dimension is also related to the growth rate. This is little surprising, considering that living things usually follow some rules with regard to resource use efficiency that lead to differences in “fitness” associated with different designs (e.g., Niklas, 1994).

Since the approach presented here is solely based on a single holistic measure it is difficult to compare it to other approaches. From the results of previous studies, we know that other structural measures, particularly those derived from existing laser‐based approaches, are also and with high levels of statistical significance sensitive to the tested treatments “neighborhood diversity” (pure vs. mixed), “growth conditions” (at gap vs. in interior), and “competition” (little to strong). However, those measures, for example, crown base height are often defined in nonmathematical terms, such as “height of the first leave‐bearing branch” in the case of crown base height. Such definitions are not easily converted to mathematical procedures that derive the measures from objective 3D data. Therefore, objectivity may be a reasonable argument for the use of the box dimension approach. If 3D data on a tree are available, which is increasingly the case in scientific studies, the computation of D_b_ is fast, straight forward, objective, and requires very little predefined settings or conditions (only the box sizes used).

Here, it is argued that the presented holistic approach is not a substitute for existing measures. It is a different approach to tree structure that shows promising relationships with physiological measures like productivity while at the same time being sensitivity to tree species, neighborhood diversity, or growing condition as shown here.

## 5 CONCLUSIONS AND OUTLOOK

The idea of the fractal dimension, and therefore also of the box dimension, is to provide a single meaningful measure of the complexity of objects. In this study, it was shown that three‐dimensional data from terrestrial laser scanning can be used to successfully derive the box dimension of trees and make use of its holistic perspective on structure, architecture or, in more general: complexity. It was shown that *D*_b_ is a meaningful measure of tree structural complexity that is not only significantly different for tree species that differ in their morphology. The measure also seems to be a powerful descriptor for external drivers of structure, such as competition strength and competition type. For Douglas‐Fir trees growing in the Pacific Northwest, it was shown that *D*_b_ is sensitive to one‐sided competition as experienced by trees growing on the edge of a gap. Furthermore, for European beech, morphological adaptations of the tree crown to different competition situations (intraspecific vs. interspecific competition) were shown to be reflected in *D*_b_. Particularly interesting is the relationship between the growth and *D*_b_ of tree individuals, as shown for beech trees in a temperate forest in Germany.

From this first study, it can be concluded that the application of fractal analysis to tree point clouds holds a so far unexplored potential to provide a deeper understanding of ecophysiological process that drives tree architecture and ultimately forest structure. *D*_b_ was shown to be an integrating measure that may provide new insights into the external drivers of tree architectural complexity and it may support a better understanding of structure‐related processes, like tree growth, in the future.

For example, one potential application of the box dimension is the analysis of the *R* ^2^ values of the linear regression. Trees with a high level of architectural self‐similarity are expected to have higher *R* ^2^ values (greater linearity) than trees that are less self‐similar.

Another example would be the analysis of cutoffs. Cutoffs are scales at which the least square fit based on a straight line is not reasonable anymore, for example, due to nonlinearity. Such cutoffs may define boundaries for extrapolation of physiological processes in trees and may support distinguishing hierarchical scales (Sugihara & May, 1990). Using the methods described here such analysis would be enabled.

## CONFLICT OF INTEREST

None declared.

## DATA ACCESSIBILITY

Data are archived on the servers of the Gesellschaft für Wissenschaftliche Datenverarbeitung mbH Göttingen.
